# Effects of Playing Angklung and Practicing Silence on Emotion, Cognition and Oxytocin Levels in Children: A Preliminary Study

**DOI:** 10.21315/mjms2021.28.3.10

**Published:** 2021-06-30

**Authors:** Margarita M Maramis, Yunias Setiawati, Nining Febriyanti, Munawaroh Fitriah, Atika, Rasyid Salim, Budi Kristianto, Nyoman Sumiati, Vista Nurasti Pradanita, Elisabet Citra Dewi, Sheila Maryam Gautama, MY Safira Nugroho, Jakobus Gerick Pantouw

**Affiliations:** 1 Department of Psychiatry, Faculty of Medicine, Airlangga University/Dr. Soetomo General Academic Hospital, Surabaya, Indonesia; 2 Department of Clinical Pathology, Faculty of Medicine, Airlangga University/Dr. Soetomo General Academic Hospital, Surabaya, Indonesia; 3 Department of Community Health, Faculty of Medicine, Airlangga University, Surabaya, Indonesia; 4 Department of Psychiatry, Faculty of Medicine, Airlangga University, Surabaya, Indonesia; 5 Department of Psychiatry, Faculty of Medicine, Widya Mandala Catholic University, Surabaya, Indonesia

**Keywords:** child well-being, silence, emotion, cognition, oxytocin

## Abstract

**Background:**

Playing angklung, an Indonesian traditional musical instrument and practicing silence have been shown to exert beneficial effects on emotion and cognition; the mechanism of such an effect possibly involve oxytocin. To date, only a few clinical and biomolecular studies have investigated the effects of playing angklung and practicing silence. This study aimed to examine the effects of playing angklung and practicing silence on human emotion and cognition and on oxytocin levels.

**Methods:**

This experimental study involved 61 Fourth Grade students from Surabaya, East Java, Indonesia. The participants were divided into two groups: the angklung intervention group and silence intervention group. The two interventions were performed for 15 min before the morning classes for 2 months. The control group attended their classes as usual. Clinical parameters, namely, emotion and cognition, as well as the saliva oxytocin levels were measured.

**Results:**

No significant differences were observed among the three groups in terms of concentration and cognitive flexibility. However, changes in oxytocin levels significantly differed among the three groups (*P* < 0.001) and the oxytocin levels were highest in the silence intervention group.

**Conclusion:**

Practicing silence significantly increased the oxytocin levels, but it did not elicit changes in cognitive function and emotion of the students.

## Introduction

Humans learn continuously throughout their lives and their learning process is influenced by various factors, including cognition and emotion. Cognitive function is crucial for processing both intrinsic and extrinsic information. The important aspects of cognitive function are concentration and cognitive flexibility. Concentration can be likened to a window through which information enter the brain. Concentration (attention) is a process of resolving, managing and eliminating disturbance or unneeded stimuli, and it involves both conscious and unconscious processes (1). A certain degree of concentration is required to facilitate a good learning process (2). Cognitive flexibility is a component of the executive function, along with inhibitory ability and working memory (3); cognitive flexibility indicates a person’s preparedness to change their mental processes in order to arrive at an appropriate response, an important ability in appropriately and efficiently responding to environmental changes. Cognitive flexibility allows a person to work efficiently, to shift from one task to another, and to determine the appropriate responses to conditions that arise. The more flexible a person’s cognition is, the better the output will be throughout their life (4). Meanwhile, emotions are feelings elicited by a stimulus. As stated by Yang (5), it is impossible to build memories, which involve complex thoughts or to make meaningful decisions without the involvement of emotions.

Children learn to develop their emotions and cognition by receiving stimulation from the outside world. One such stimulus is music, that is, either listening or playing music. Music is a universal art form that exists in every culture; it consists of various elements, including rhythm, melody, harmony, counterpoint and orchestration (6). Learning music enhances the intelligence by 35% compared with learning computers and crafts; it also improves emotional intelligence (7). Several neuroscience studies have observed an enhanced brain development in children due to musical training, specifically in the verbal domain of working memory, pronunciation accuracy in second language, reading ability and executive functions. Moreover, some researchers have found a difference in the brain development of musicians and non-musicians. Neuroimaging studies have revealed changes in the brain plasticity of adult musicians, although the extent of the effect of music on brain plasticity relative to that of other factors as well as the effects of music on biological markers remain unclear (3, 8).

Various kinds of musical instruments exist worldwide and one of these instruments is angklung. Angklung is a traditional bamboo musical instrument from the Sunda region in West Java, Indonesia. It is a rhythmic, multi-tonal instrument that is played by shaking. The Indonesian Department of Education and Culture designated angklung as an official educational musical instrument through the Decree No. 082/1968 dated 23 August 1968. This musical instrument requires its players to be cooperative, disciplined, skilful and responsible in playing pieces of music. On 10 September 1968, Daeng Soetigna, a music teacher who modernised angklung and is considered the father of modern angklung music, symbolically handed angklung to the UNICEF. At the same time, he introduced his angklung education motto known as the 5M, which stands for *mudah* (easy), *murah* (cheap), *menarik* (interesting), *mendidik* (educational) and *massal* (mass). Angklung was also included in the representative list of the Intangible Cultural Heritage of Humanity on 18 November 2010 by the UNESCO (7). The positive effects of playing angklung have previously been reported, such as increased creativity in students (9), indirectly improved emotional regulation among university students (10), improved gross motor skills in children (11), improved emotional and social development in mentally disabled children (12) and enhanced emotional intelligence in students who play angklung as their extracurricular activity (7). In the elderly, playing angklung helps reduce loneliness (13) and it improves the quality of life of those who live in nursing homes (14).

Similar to active stimulation, practicing moments of silence offers several benefits. Learning has been found to be more effective when done in a silent environment. A noisy environment may increase cortisol levels, which in turn may be reduced by silence. Moreover, routine practice of momentary silence is beneficial to individuals. According to Fitzgerald (15), when the brain is in a state of silence, there is an increase in neuronal cell growth. Other activities, such as meditation, which also lead to silence, increases awareness and self-regulation (16, 17). An increase in self-regulation is associated with an increase in grey matter volume in the anterior cingulate cortex. In the prefrontal cortex, an increase in grey matter volume may benefit other functions, such as emotional regulation, planning and problem solving (17), and it may also benefit hippocampus development (18).

Oxytocin is a hormone produced by the hypothalamus and is secreted by the posterior pituitary gland. It is a peptide consisting of nine amino acids (Cys–Tyr–Ile–Gln–Asn–Cys–Pro–Leu–Gly–NH_2_) with a molecular mass of 1,007.19 g/mol or 1,007 Da (1 IU oxytocin = 1.68 μg pure peptide). It plays a role in uterine contraction during childbirth and in milk secretion during breastfeeding. Oxytocin secretion depends on the electrical activity of neurons in the hypothalamus, wherein oxytocin is released when the cells are excited. Oxytocin acts as a chemical messenger in the brain, and it plays a role in social behaviours, such as sexual arousal, recognition, trust, anxiety and mother-child bond; thus, it is known as the love hormone or the cuddle chemical (19). Oxytocin facilitates emotional empathy irrespective of culture and gender (20). Studies have found that oxytocin is associated with social cognition (21) and meta-mood (22). It has also been reported to be involved in stress reduction and well-being (23–25). An increase in oxytocin levels, along with improved mood, has been observed following mindfulness sessions (26, 27). Therefore, we hypothesised that the emotional and cognitive effects of playing angklung or practicing silence may involve oxytocin; hence, this intervention might affect the oxytocin levels.

To date, the effects of playing angklung and of practicing silence on emotion, cognition and oxytocin levels have not been studied. This study aimed to examine the impacts of playing angklung and practicing silence on mood, cognition and oxytocin levels in elementary school children who are still undergoing periods of growth and brain development. If positive results are obtained, then these activities may be proposed as an additional activity for students.

## Methods

This experimental study was conducted in Sekolah Dasar Negeri Klampis Ngasem I, an elementary school in Surabaya, East Java, Indonesia. The inclusion criteria were as follows: students in the Fourth Grade; students without special needs; students willing to participate in this study and students whose parents signed an informed consent form. The exclusion criteria were as follows: students with mental retardation and those who were ill or undergoing medication during the study period. The dropout criteria were as follows: the subject did not participate in the intervention for ≥ 10% of the total duration (8 weeks) of the intervention; the subject was on academic leave and the subject withdrew from the study.

The one-stage cluster random sampling by class was employed to determine the sample size for the angklung intervention, silence intervention and control groups. For each group, clinical parameters were examined. The number of samples for the oxytocin measurement for each group was six, which was determined using the formula for hypothesis testing involving the comparison of two populations (Equation 1) (28). Since the dropout rate was estimated to be 50%, each group should include a minimum of nine subjects, who were then randomly selected. In the angklung intervention group, the subjects were taught to play various national and regional songs that uses all available notes for 15 min every day before their morning classes for 8 weeks. In the silence intervention group, the subjects were taught to calm themselves and remain silent for 15 min every day before their classes for 8 weeks.

(1) n≥(Z1-α2+Z1-α)2+(σ12+σ22r)(μ1-μ2)2

Equation 1. Calculation of the minimum sample size for the comparison of two means using the following parameters: *α* = 0.05, *β* = 0.2, *μ*_1_ = 49.28, *σ*_1_ = 11.02, *μ*_2_ = 64.99, *σ*_1_ = 11.26, *r* = 1.

### Clinical Examination Instruments

Cognitive functions, which are important for processing both external and internal information, involve many domains. The most important domains are concentration and cognitive flexibility. Concentration is the ability to focus one’s attention or thoughts toward a particular matter and it is measured using the trail making tests A and B (TMT-A and TMT-B). Cognitive flexibility is the ability to think of alternatives and to change plans, and it is measured using the Wisconsin card sorting test (WCST). Emotional function is a burst of feelings that develops and subsides within a short period. Emotional parameters were examined using the positive and negative affect scale (PANAS) questionnaire for children (29, 30).

TMT-A and TMT-B are commonly used in neuropsychological assessments. This test is simple and may be used to assess various cognitive processes, including attention, visual search and scanning, sequencing and shifting, psychomotor speed, abstract thinking, flexibility, ability to carry out and modify plans, and the ability to maintain two thoughts simultaneously. These tests can be used for subjects aged 7–89 years old and they have been found suitable for younger children (31, 32). These tests have also been used in a local Indonesian population (33). These tests consist of 25 circles containing numbers or letters. In the TMT-A, the circles contain numbers from 1 to 25 and the subjects were asked to connect each circle in their correct sequence. In the TMT-B, some circles contain numbers [1–13] and the remaining circles contain letters (A–L); the subjects were asked to connect the circles containing numbers and letters alternately in their correct sequence (example: 1-A-2-B-3-C). The subjects must complete the task as fast as possible without lifting the pencil from the test paper. The time the subjects needed to complete these tasks was recorded. If the subject makes a mistake, the examiner, without stopping the time, immediately notifies the subject and allows them to correct the mistake made. The subjects were scored based on the amount of time they needed to complete the test. The shorter the time the subject needed to complete the test, the better their concentration (34).

The WCST was used to measure neuropsychological functions, abstract thinking, cognitive flexibility and executive function. This test can be used on subjects aged 5–89 years old and it requires 10 min–15 min to complete; it has also been found to be appropriate for school-age children (35, 36). The validity of the WCST to measure cognitive flexibility has been tested internationally. The test has also been used in an Indonesian population (37, 38). The subjects were scored based on the number of mistakes they made (0%–100%) with scores ≤ 13% considered to indicate normal condition. The subjects were given 60 cards to sort; each card bears a symbol (triangle, star, plus or circle), which varied in terms of colour (red, green, yellow and blue) and number [1–4]. The combination of symbols, colours and numbers were randomised and no two cards were identical. Four stimulus cards were placed on the table (target cards): one red triangle, two green stars, three yellow plus signs and four blue circles. Before the test began, the examiner determined a sorting pattern (i.e. based on colour, shape or number) that was unknown to the subject. During the test, the examiner would change the sorting pattern without telling the subject. The subject was tasked to guess the sorting pattern by taking one card from the pile of cards to be sorted and placing it under a stimulus card (e.g., if the subject made a guess that the sorting pattern was based on colour, the subject would match the colour of the card they picked from the pile with the colour of the stimulus card). Each time the subject put a card down, the examiner would comment ‘right’ or ‘wrong’ as feedback. When the examiner changed the sorting pattern, the subject must guess the new pattern. The test ended when all cards in the pile were used. A lower score indicated fewer mistakes made and thus a better cognitive flexibility (39–42).

The PANAS was developed as the operationalisation of the orthogonal dimensions of positive affect (now called positive activation [PA]) and negative affect (negative activation [NA]) that emerged from the analyses of Zevon and Tellegen (43) and Watson and Tellegen (44). Since its publication, the PANAS has become one of the most widely used measures of affect. It consists of 20 items, 10 for the PA scale (e.g., interested and excited) and 10 for the NA scale (e.g., distressed and upset). Each item is accompanied by a 5-point scale, where 1 indicates ‘very slightly or not at all’ and 5 indicates ‘extremely’. PANAS-P indicates a positive emotion and PANAS-N indicates a negative emotion. PANAS demonstrates a high reliability and validity for various samples and time frames (e.g., for the past month) and has been translated in many languages. PANAS-C, which was developed by Laurent et al. (45), was designed specifically for children aged 7–14 years and consists of 27 items (12 items PANAS-P and 15 items PANAS-N). For both tools, the higher the score, the more intense the positive or negative emotion being experienced (29, 30, 46, 47). The reliability and validity of PANAS-C have been previously reported (45, 48, 49). The PANAS-C was used in this study; it was translated from English to Indonesian and back to English by an expert translator to ensure its validity and reliability for use in the Indonesian population.

### Protein Biomarker Measurement

Saliva oxytocin levels were measured with a human oxytocin ELISA kit (ADI-901-153; Enzo Life Sciences, Farmingdale, NY, USA).

## Sample Collection

### Fasting Procedures

The subjects were recommended to avoid eating, drinking and brushing their teeth prior to the saliva collection (50, 51).

## Saliva Collection

Saliva samples were collected in the morning using the passive drooling method. The participants were instructed to tilt their head forward and pool their saliva in their mouths. When a sufficient amount of saliva was pooled, the participants were asked to drool into a cryovial (Salimetrics, Carlsbad, CA, USA) until 1.5 mL of saliva was collected. The samples were stored in a cooling bag (~8 °C) immediately after collection, transferred to the laboratory and centrifuged at 1650×g at 4 °C for 10 min. Samples were aliquoted and stored at −80 °C within 1 h after collection (52).

## Enzyme-Linked Immunosorbent Assay (ELISA) Kit

Salivary oxytocin was measured using a 96-plate commercial OT-ELISA kit (Enzo Life Sciences) according to the manufacturer’s instructions. The optical density of the samples and standards was measured by a microtiter plate reader (Human Diagnostic, Germany). The oxytocin concentrations were calculated according to a relevant standard curve. Saliva was not extracted in this experiment similar to the approach employed in some studies (53).

### Statistical Analysis

Normality tests for the changes in PANAS-P, PANAS-N, TMT-A, TMT-B and WCST scores and the changes in oxytocin levels in the angklung intervention, silence intervention and control groups were performed using the Shapiro-Wilk test. A homogeneity test was performed using the Levene’s test. Differences between groups were tested using ANOVA.

## Results

Of the 61 students, 60% were male. Table 2 presents the gender distribution in each group and Table 3 presents the distribution of the clinical parameters and saliva oxytocin levels.

The normality tests using the Shapiro-Wilk test showed a normal distribution and homogeneity for the parameter changes in PANAS-P, PANAS-N, TMT-A, TMT-B, and WCST (Table 1). The ANOVA test showed that there are no significant differences among the groups in terms of the emotional and cognitive parameters (Table 3).

The Shapiro-Wilk normality test showed a normal distribution of oxytocin levels within the groups, except for the control group (Table 1). Moreover, the Levene’s test for homogeneity of variance showed equal variances (*F*[3, 36] = 2.16, *P* = 0.11). The ANOVA test performed to examine the differences in the changes in oxytocin levels between groups found a significant difference among the three groups (Table 3). The Tukey post-hoc test, which determines which group differences are significant, showed that the differences in oxytocin levels between the angklung and silence intervention groups (*P* = 0.112) and between the angklung intervention and control groups (*P* = 0.056) were not significant, whereas those between the silence intervention and control groups (*P* < 0.001) were significant.

## Discussion

Humans are social beings equipped with cognitive functions, which are more complex than those of any other organism and which form the foundation of all aspects of daily living, including social and all other aspects of our lives. The aforementioned cognitive function is taught by parents or caretakers at home, as well as by teachers in school. In terms of its functioning, cognition is influenced by emotions that likely affect the learning process in school. In terms of neurobiology, memory formation, complex thinking or decision making involve one’s emotions.

### Emotion

Emotion is a complex psychological and physiological state that gives meaning to an event. Functional neuroimaging analysis has shown that unlike other psychological functions, emotion is relatively unorganised and it affects all aspects of human cognition. Emotional disorders, especially those related to stress, may cause damage to various human functions (54). Emotions affect the logical reasoning process irrespective of a task (55). As previously reported, children who were under stressful and threatening conditions had a limbic system that was activated even before their prefrontal cortex had the chance to evaluate the stimulus. However, the prefrontal cortex can perform learning and problem solving only in a non-hyperarousal state (55). Therefore, the ability to regulate one’s emotion is crucial.

Emotion regulation is essential in a person’s life. Educators are expected to be capable of teaching their students the aforementioned emotion regulation skills so that their students can effectively resolve their social, moral and cognitive problems. However, educators often lack an understanding of how emotions largely affect the learning process in school (5). In this study, the PANAS-C scores showed that playing angklung, practicing silence and the control condition did not significantly increase the students’ positive emotions, although negative emotions were decreased. This finding may have been affected by the examination method; the assessment was conducted *en masse* (i.e. involving the entire class) and this approach may have caused biases in the students’ understanding of a given word even though the meaning of each word was explained beforehand. Nevertheless, emotional screening of the students was done periodically to determine their emotional states longitudinally given that the school teachers were less knowledgeable about the effects of emotional problems that possibly make it difficult for students to understand their lessons (5). Situational effects during the tests, such as the environmental and ambient atmospheric conditions, which constantly change, may have also influenced the results, especially that the pre- and post-test could not be done under the same exact condition as the tests were conducted on different days. Moreover, the students’ schedules vary each day, wherein they do different activities, such as physical education, flag ceremonies and extracurricular activities, all of which may influence the student’s feelings. The intelligence and cultural background of a child may also affect their understanding and expression of their own feelings. Cross-cultural research on six basic emotions, namely, sadness, fear, disgust, anger, surprise and happiness, showed a difference in the cognitive and affective perception of emotion between American Canadians and Chinese Koreans. Thus, positive emotion does not always elicit a positive impact and vice versa (57).

### Cognitive Function

Cognitive function, especially concentration, plays an important role in the input, selection, retention, division and storage of information. Information is subsequently processed with cognitive flexibility, which facilitates broader considerations so that appropriate decisions can be made; also, cognitive flexibility limits ruminations that lead to incorrect decisions. Rumination is defined as the unintentional repetitive thinking beyond control, which may occur in cases of cognitive impairment, specifically impairment of the executive function, and it may be affected by mood. People who ruminates cannot inhibit their ruminating thoughts, and their attention is fixated only on one thought (58). Cognitive flexibility is an essential ability in adapting to an ever-changing environment (59). A good cognitive flexibility will lead to better outputs in life, for instance, children become better at reading and become more resilient to life challenges or stresses, adults become more creative, and the elderly experience a better life quality (4). Cognitive flexibility is the ability to shift from one thought or from a train of thoughts as an adaptive reaction toward stimuli, and it is broadly defined as the ability to adapt behaviours in response to changes in the environment. In neuroscience, cognitive flexibility is known as attention switching, cognitive shifting, mental flexibility, set shifting and task switching. Along with attention control and inhibition system, cognitive flexibility is a critical component of executive function (59, 60). Together with other executive functions, including inhibition, attentional control and working memory, cognitive flexibility facilitates complex skills, such as goal-directed planning, problem solving and deliberate learning (60).

This study did not find significant changes in concentration and cognitive flexibility in the three groups. However, the differences in TMT-A and WCST results were close to reaching a statistical significance. The investigated clinical parameters require a longer treatment period to display changes compared with bio-molecular markers, in this case oxytocin. This inconsistency may have been caused by situational factors during examination as mentioned above. The clinical examination of children requires an environment that is free from the influences of other variables so that the results can reflect the effects of the treatments; however, this requirement was not met in this study.

Another possible contributing factor was that the school where this study was conducted is a pilot school, wherein many pilot activities are done, which may have overloaded the students. Moreover, the students may have had other stressors in their home, friends or studies. The concept of allostatic load in children can be applied to prevent excessive positive stimulation, which may become counterproductive. Stressors are either detrimental or protective factors (61, 62); therefore, each child must be examined thoroughly and evaluated individually. From childhood up to pre-adolescence, the brain experiences a growth period wherein there is an increase in grey matter volume; during adolescence, the grey matter volume decreases while the white matter volume increases (63). Thus, during childhood, children must be given appropriate positive stimulation to allow optimum growth. Stimulation may become a prolonged stressor when it exceeds a child’s limits, which may lead to health and mental problems (64).

In the WCST test for cognitive flexibility, the decrease in the number of mistakes made was greater in the angklung intervention group than in the silence intervention group, although the difference was not statistically significant.

### Oxytocin

Oxytocin is a hormone that modulates the sensory system and plays a role in the response to social stimulation in nematodes and humans. Moreover, it is important in childbirth, in the display of a nurturing behaviour and in mothers’ behaviour toward their infants. During the infancy period, a positive correlation exists between parent–infant contact and oxytocin levels. Oxytocin modulates social information, increases social sensitivity, and modulates reactivity to stressors. In all species, oxytocin receptors are concentrated in the cholinergic areas involved in visual and auditory processes, specifically the basal nucleus of Meynert that coordinates neuronal activities in the amygdala and cortex (65, 66). Playing angklung requires the processing of visual and auditory stimuli, whereas practicing silence induces calmness and relaxation. Both activities are hypothesised to increase oxytocin levels and, improve emotional and cognitive functions.

In this study, significant changes in oxytocin hormone levels were observed. The oxytocin levels in the silence intervention group were significantly increased compared with those in the control group, but such an increase did not result in significant changes in the clinical parameters. These inconsistent results between the clinical parameters and biomolecular markers are possibly due to the changes in the biomolecular markers, in this case neurohormones, prior to the onset of clinical changes. It is possible that practicing silence for a prolonged period may increase the biomolecular and clinical markers more significantly. Oxytocin may play a role in empathy processes, social cognition and social decision making as well as in emotional regulation. Whether an increase in oxytocin precedes, independent of, or secondary to social stimulation effects remain unclear. Moreover, changes in oxytocin levels depend on the context of the study and on individual variations, including gender, personality and life experiences (67). The experimental treatment may need to be performed repeatedly without exceeding the allostatic load in order to achieve clinical significance.

By contrast, a slight decrease in oxytocin levels was observed in the angklung intervention group, whereas a considerable decrease was observed in the control group. This result may have also been influenced by the level of complexity of playing angklung, which requires effort in understanding codes and in memorising and playing coded notes, ultimately requiring greater concentration than practicing silence. Therefore, each child may have their own difficulty factors. The greatest decrease in oxytocin level was observed in the control group, and this result was obtained possibly because this non-intervention group have had so much activities that they had no time for leisure activities. Oxytocin induces anti-stress-like effects, such as reduction of blood pressure and cortisol levels, as well as exerts an anxiolytic-like effect and stimulates various types of positive social interaction (68). Thus, practicing silence is an option for relaxation activities as it can increase the oxytocin levels. Unlike practicing silence, playing angklung may have no effect on emotional changes and social cognition due to the decrease in oxytocin levels. However, the desired effect may eventually be observed when the students have gotten used to and enjoy playing the musical instrument.

The results of this study can be optimised by considering the following to address the current limitations: i) use a larger sample size, as this work is a preliminary study involving a limited number of subjects, which may have influenced the observed effect size; ii) employ fewer examiners to avoid inter-rater differences when examining a large number of subjects, who must be tested at the same time before and after a 2-month intervention and iii) extend the intervention period as a prolonged intervention may yield more tangible changes; the intervention period of 2 months is apparently insufficient to observe changes in clinical parameters, namely, cognitive function and emotion.

## Conclusion and Future Directions

To the best of our knowledge, this is the first study to demonstrate the effect of playing angklung and of practicing silence on emotion, cognition and oxytocin levels. The oxytocin levels were significantly higher in the silence group than in the control, although the increase in oxytocin levels did not result in a change in clinical parameters, namely, concentration and cognitive flexibility. Moreover, playing angklung for 15 min each day for 2 months did not exhibit a significant efficacy. A longer intervention period within an environment that is free from overstimulation may be needed in order to observe the optimum effects of the intervention.

## Figures and Tables

**Table 1:** Normality and homogeneity tests

Change (Δ)	Playing angklung	Practicing silence	Control group	Homogeneity test
			
	Sig.*	Sig.*	Sig.*	Sig.
PANAS-P	0.788	0.365	0.455	0.255
PANAS-N	0.724	0.233	0.512	0.729
TMT-A	0.051	0.888	0.341	0.453
TMT-B	0.154	0.054	0.548	0.458
WCST	0.989	0.653	0.540	0.828
Oxytocin levels	0.357	0.051	0.041**	–

Note:

* Saphiro-Wilk normality test;

** *P* < 0.05

**Table 2:** Subject gender distribution

		Group			Total *n* (%)

		Playing angklung *n* (%)	Practicing silence *n* (%)	Control group *n* (%)	
Gender	Male	12 (60.0)	9 (42.9)	11 (55.0)	32 (52.5)
	Female	8 (40.0)	12 (57.1)	9 (45.0)	29 (47.5)

Total		20 (100.0)	21 (100.0)	20 (100.0)	61 (100.0)

**Table 3:** Average results of clinical parameters examination and saliva oxytocin level from playing angklung, practicing silence and control groups

	Angklung				Silence				Control				Sig.

	Baseline mean± SD	Δ mean±SD	95% CI		Baseline mean±SD	Δ mean±SD	95% CI		Baseline mean±SD	Δ mean±SD	95% CI		
		
			Lower	Upper			Lower	Upper			Lower	Upper	
PANAS-P	50.9±5.22	−2.35±5.04	−4.71	0.010	48.9±9.87	−1.00±7.25	−4.49	2.49	46.0±6.77	−2.85±6.84	−6.05	0.35	0.653
PANAS-N	29.4±9.38	5.20±10.94	0.08	10.32	28.8±7.63	4.89±7.71	1.18	8.61	32.7±9.49	2.00±10.83	−3.07	7.07	0.540
TMT-A	56.6±17.5	−2.05±22.67	−12.98	8.88	58.5±17.2	−11.67±13.66	−17.89	−5.45	68.0±25.2	−17.40±22.04	−27.71	−7.09	0.058
TMT-B	99.0±47.1	−17.53±27.71	−30.88	−4.17	113±38.0	−34.67±31.89	−49.18	−20.15	117±40.1	−36.05±29.68	−49.94	−22.16	0.108
WCST	36.2±16.8	−7.35±11.67	−13.35	−1.35	30.7±13.3	−1.75±10.70	−6.62	3.13	31.0±13.7	−9.47±12.10	−15.30	−3.64	0.097
Oxytocin levels	93.7±35.0	−22.33±39.20	−50.37	5.71	82.8±41.8	35.81±52.77	−1.94	73.56	173±117	−88.65±83.28	−148.22	−29.078	< 0.001**

Notes:

* ANOVA: *F* = 8.37, *P* < 0.001; Tukey post-hoc: Silence–Control, *P* < 0.001; Angklung–Control, *P* = 0.056; Angklung–Silence, *P* = 0.112

## References

[1] Sternberg RJ, Sternberg K (2017). Cognitive psychology.

[2] Malone S (2003). Learning about learning: an A–Z of training and development tools and techniques.

[3] Sachs M, Kaplan J, Der Sarkissian A, Habibi A (2017). Increased engagement of the cognitive control network associated with music training in children during an fMRI stroop task. PLoS One.

[4] Dajani DR, Uddin LQ (2015). Demystifying cognitive flexibility: implications for clinical and developmental neuroscience. *Trends Neurosci*.

[5] Yang M (2015). Emotions, learning and the brain.

[6] Thomson V (1957). Introduction to Robert Erickson. The structure of music: a listener’s guide: a study of music in terms of melody and counterpoint.

[7] Pudjiastuti Endang, Sari Yunita, Suryani Emotional intelligence of the students participating in an angklung extracurricular activity.

[8] Miendlarzewska EA, Trost WJ (2014). How musical training affects cognitive development: Rhythm, reward and other modulating variables. Front Neurosci.

[9] Pramana I (2015). Peran ansambel angklung dalam mengembangkan kreativitas siswa di SMP Negeri 1 Kaliori Kabupaten Rembang. Masters’ diss.

[10] Ira Anzaina RDP (2015). Regulasi emosi pada mahasiswa fakultas psikologi universitas padjadjaran yang bermain angklung [Master’s diss].

[11] Marlina, Ali M, Halida (2013). Peningkatan kemampuan motorik kasar anak melalui permainan alat musik angklung pada anak usia 5–6 tahun. Khatulistiwa.

[12] Umar Djani M, Warnandi N, Nurhaeni H (2009). Pengaruh pembelajaran musik angklung. JAffl_ Anakku.

[13] Ariani DR, Hartiah Haroen S (2014). Pengaruh terapi musik angklung terhadap kesepian pada lansia di Rumah Perlindungan Sosial Tresna Werdha Garut. *J Keperawatan Padjadjaran*.

[14] Komariyah L (2016). Pengaruh angklung terhadap kualitas hidup wanita lanjut usia. J Pendidikan Keperawatan Indonesia.

[15] Fitzgerald E (2016). Neuroscience reveals what happens to the brain during silence: benefits of meditating and listening to nothing.

[16] Salthouse TA (2011). What cognitive abilities are involved in trail-making performance?. Intelligence.

[17] Tang Y, Ma Y, Wang J, Fan Y, Feng S, Lu Q (2007). Short-term meditation training improves attention and self-regulation. Proc Natl Acad Sci USA.

[18] Kirste I, Nicola Z, Kronenberg G, Walker TL, Liu RC, Kempermann G (2015). Is silence golden? effects of auditory stimuli and their absence on adult hippocampal neurogenesis. Brain Struct Funct.

[19] Hormone Health Network (2018). What is oxytocin? Hormone Health Network, Endocrine Society.

[20] Geng Y, Zhao W, Zhou F, Ma X, Yao S, Hurlemann R, Becker B (2018). Oxytocin enhancement of emotional empathy: generalization across cultures and effects on amygdala activity. Front Neurosci.

[21] Rault JL, van den Munkhof M, Buisman-Pijlman FTA (2017). Oxytocin as an indicator of psychological and social well-being in domesticated animals: a critical review. *Front Psychol*.

[22] Ebner NC, Horta M, Lin T, Feifel D, Fischer H, Cohen RA (2015). Oxytocin modulates meta-mood as a function of age and sex. Front Aging Neurosci.

[23] Matsushita H, Latt HM, Koga Y, Nishiki T, Matsui H (2019). Oxytocin and stress: neural mechanisms, stress-related disorders, and therapeutic approaches. Neuroscience.

[24] Ito E, Shima R, Yoshioka T (2019). A novel role of oxytocin: oxytocin-induced well-being in humans. Biophys Physicobiology.

[25] Ishak WW, Kahloon M, Fakhry H (2011). Oxytocin role in enhancing well-being: a literature review. J Affect Disord.

[26] Bellosta-Batalla M, del Carmen Blanco-Gandía M, Rodríguez-Arias M, Cebolla A, Pérez-Blasco J, Moya-Albiol L (2020). Brief mindfulness session improves mood and increases salivary oxytocin in psychology students. Stress Heal.

[27] Lipschitz DL, Kuhn R, Kinney AY, Grewen K, Donaldson GW, Nakamura Y (2015). An exploratory study of the effects of mindbody interventions targeting sleep on salivary oxytocin levels in cancer survivors. Integr Cancer Ther.

[28] Lameshow S, Hosmer DW, Klar JLS (1990). Adequacy of sample size in health studies.

[29] Crawford JR, Henry JD (2010). The positive and negative affect schedule (PANAS): construct validity, measurement properties and normative data in a large non-clinical sample. Br J Clin Psychol.

[30] Hughes AA, Kendall PC (2009). Psychometric properties of the positive and negative affect scale for children (PANAS-C) in children with anxiety disorders. Child Psychiatry Hum Dev.

[31] Leòn-Carriòn J (1989). Trail making test scores for normal children: normative data from Spain. Percept Mot Skills.

[32] Reitan RM (1971). Trail making test results for normal and brain-damaged children. Percept Mot Skills.

[33] Estiasari R, Aryanto I, Lee S, Pramana S, Djauzi S, Price P (2020). Determinants of cognitive health in Indonesian HIV patients beginning antiretroviral therapy. J Neurovirol.

[34] Lezak MD, Howieson DB, Loring DW (2004). Neuropsychological assessment.

[35] Rosselli M, Ardila A (1993). Developmental norms for the Wisconsin card sorting test in 5- to 12-year-old children. Clin Neuropsychol.

[36] Romine CB, Lee D, Wolfe ME, Homack S, George C, Riccio CA (2004). Wisconsin card sorting test with children: a meta-analytic study of sensitivity and specificity. Arch Clin Neuropsychol.

[37] Nugroho MYS, Daeng BH, Waworuntu GL (2019). Cognitive flexibility and problem-solving in patients with bipolar disorder. Biomol Heal Sci J.

[38] Noor YB, Mahajuddin MS (2015). Efek terapi remediasi kognitif terhadap perbaikan fungsi kognitif pasien skizofrenia dengan terapi standar yang rawat jalan di RSJ Menur Surabaya.

[39] Feldstein SN, Keller FR, Portman RE, Durham RL, Klebe KJ, Davis HP (1999). A comparison of computerized and standard versions of the Wisconsin card sorting test. Clin Neuropsychol.

[40] Kongs S, Thompson L, Iverson G, Heaton R (2000). Wisconsin card sorting test-64 card version. Psychological Assessment Resources.

[41] Kohli A, Kaur M (2006). Wisconsin card sorting test: normative data and experience. Indian J Psychiatry.

[42] Greve KW (2001). The WCST-64: a standardized short-form of the Wisconsin card sorting test. Clin Neuropsychol.

[43] Zevon MA, Tellegen A (1982). The structure of mood change: an idiographic/nomothetic analysis. J Pers Soc Psychol.

[44] Watson D, Tellegen A (1985). Toward a consensual structure of mood. Psychol Bull.

[45] Laurent J, Catanzaro SJ, Joiner TE, Rudolph KD, Potter KI, Lambert S (1999). A measure of positive and negative affect for children: scale development and preliminary validation. Psychol Assess.

[46] Watson D, Clark LA (1994). The PANAS-X manual for the positive and negative affect schedule — expanded form Iowa Research Online.

[47] Wróbel M, Finogenow M, Szymańska P, Laurent J (2019). Measuring positive and negative affect in a school-based sample: a polish version of the PANAS-C. J Psychopathol Behav Assess.

[48] Ebesutani C, Okamura K, Higa-McMillan C, Chorpita BF (2011). A psychometric analysis of the positive and negative affect schedule for children-parent version in a school sample. Psychol Assess.

[49] Laurent J, Joiner TE, Catanzaro SJ (2011). Positive affect, negative affect, and physiological hyperarousal among referred and nonreferred youths. Psychol Assess.

[50] Salimetrics (2015). Saliva collection and handling advice.

[51] Spielmann N, Wong D (2011). Saliva: diagnostics and therapeutic perspectives. Oral Dis.

[52] Vrijen C, Schenk HM, Hartman CA, Oldehinkel AJ (2017). Measuring BDNF in saliva using commercial ELISA: results from a small pilot study. Psychiatry Res.

[53] Yuhi T, Kyuta H, Mori H-a, Murakami C, Futuhara K, Okuno M (2017). Salivary oxytocin concentration changes during a group drumming intervention for maltreated school children. Brain Sci.

[54] Fin G (2016). Stress: concepts, cognition, emotion, and behavior handbook of stress.

[55] Jung N, Wranke C, Hamburger K, Knauff M (2014). How emotions affect logical reasoning: evidence from experiments with mood-manipulated participants, spider phobics, and people with exam anxiety. Front Psychol.

[56] Streeck-Fischer A, van der Kolk BA (2000). Down will come baby, cradle and all: diagnostic and therapeutic implications of chronic trauma on child development. *Aust New Zeal J Psychiatry*.

[57] An S, Ji LJ, Marks M, Zhang Z (2017). Two sides of emotion: exploring positivity and negativity in six basic emotions across cultures. Front Psychol.

[58] Brinker JK, Campisi M, Gibbs L, Izzard R (2013). Rumination, mood and cognitive performance. Psychology.

[59] Gabrys RL, Tabri N, Anisman H, Matheson K (2018). Cognitive control and flexibility in the context of stress and depressive symptoms: the cognitive control and flexibility questionnaire. Front Psychol.

[60] Legare CH, Dale MT, Kim SY, Deák GO (2018). Cultural variation in cognitive flexibility reveals diversity in the development of executive functions. Sci Rep.

[61] McEwen BS (1998). Protective and damaging effects of stress mediators. N Engl J Med.

[62] McEwen BS (2003). Mood disorders and allostatic load. Biol Psychiatry.

[63] Uytun MC (2018). Development period of prefrontal cortex [Internet]. Psychology.

[64] Rogosch, Fred, Dackis M (2012). Child maltreatment and allostatic load: consequences for physical. Dev Psychopathol.

[65] Scatliffe N, Casavant S, Vittner D, Cong X (2019). Oxytocin and early parent-infant interactions: a systematic review. Int J Nurs Sci.

[66] Young LJ (2015). Oxytocin, social cognition and psychiatry. Neuropsychopharmacology.

[67] Di Simplicio Martina (2016). Oxytocin and emotion processing. J Psychopharmacol.

[68] Uvnäs-Moberg K, Petersson M (2005). Oxytocin, a mediator of anti-stress, well-being, social interaction, growth and healing. Z Psychosom Med Psychother.

